# Strain Measurement Technology and Precision Calibration Experiment Based on Flexible Sensing Fiber

**DOI:** 10.3390/s24123811

**Published:** 2024-06-13

**Authors:** Bin Chen, Jun Yang, Ang Li, Min Zhang, Jin Li, Zhao Wang

**Affiliations:** National Key Laboratory of Intense Pulsed Radiation Simulation and Effect, Northwest Institute of Nuclear Technology, Xi’an 710024, China; chenbin1@nint.ac.cn (B.C.); liang@nint.ac.cn (A.L.); zhangmin@nint.ac.cn (M.Z.); lijin@nint.ac.cn (J.L.); wangzhao@nint.ac.cn (Z.W.)

**Keywords:** FBG, OFDR, strain calibration, repeated loading and unloading, strain jump

## Abstract

As the basic application of fiber optic sensing technology, strain measurement accuracy as a key index needs to be further calibrated and analyzed. In this paper, accuracy calibration experiments and the related analyses of two fiber-optic sensing technologies, the fiber-optic grating (FBG) and optical frequency domain reflectometry (OFDR), are carried out using a standard beam of equal strength and a mature resistive strain gauge (ESG). The fiber-optic single-point strain data for loading and unloading changes of the beams of equal strength show good continuity and linearity, with good cyclic stability, and the error in the strain test data is less than 2% after repeated loading. At the same time, using finite element theory to analyze the data and using the measured data error within 5%, a good strain test curve linearity is achieved and R2 is better than 0.998. After repeated loading and unloading tests, it is verified that the fiber grating and the distributed optical fiber in the strain test have good stability in repeatability accuracy. The calibration experiments and data analysis in this paper further illustrate the three sensing technologies in determining the strain test accuracy and the advantages and disadvantages of the indicators, and the development of the fiber optic sensing technology application provides basic technical support.

## 1. Introduction

In the process of designing, manufacturing, and applying critical structural components, it is necessary to test and evaluate the comprehensive performance of their materials and structures, such as the distribution of internal and external stresses and deformations of structural components under certain conditions of loading, temperature, etc. [1,2,3]. Typically, the stress can be obtained by monitoring the strain generated in the elastic range of the member material [4]. Through strain testing, the degree and cause of structural deformation can be analyzed, which is of great significance to the quality monitoring of structural components.

I In recent years, a variety of high-precision strain sensors have emerged in the field of sensing and testing, specifically including two categories of contact and non-contact. After years of development, resistive strain gauges have become a more mature measurement method, and as the earliest measurement technology applied in the field of strain monitoring, with a wide range of characteristics such as simple testing and reliability, it has been widely used in a variety of engineering strain testing, commonly used in the calibration of other new sensors [5,6]. However, at the same time, the use of strain gauges as point sensors in the large-scale range sensing and monitoring of the fabrication structure is complex, and under high temperature and high humidity, electromagnetic interference and other complex environments are not applicable [7,8]. With the increase in the complexity of testing needs, fiber optic sensing technology has accelerated in development. The Fiber Bragg Grating (FBG) is one of the most representative sensors in fiber optic sensors and is resistant to electromagnetic interference, corrosion, and other complex environmental factors. Multiple gratings in series can be used to carry out quasi-distributed sensing measurements relatively easily. In recent years, with the further in-depth research on FBG sensors, the application of FBG sensors has expanded from strain and temperature to various fields. By characterizing FBGs into different types, we can extend them to other parameter measurements, such as acceleration, displacement, and pressure. These are the hot application fields of grating sensors at present [9,10].

Based on the distributed sensor technology of optical frequency domain reflection (OFDR), the backscattered Rayleigh signal on the optical fiber link is retrieved through the linear swept light source combined with the coherent detection technology, and the strain and temperature sensing test with high precision and high spatial resolution is realized. Using conventional single-mode optical fiber as sensor, it is easy to realize large-scale deployment and realize point-to-point high-precision and high-resolution distributed sensing, which is often used in the fields of structural health detection, nondestructive testing of composite materials, battery temperature testing and so on in civil engineering. With the advantages of OFDR technology, it provides a new idea for the design of various sensing test schemes [11,12]. At present, with the development of FBG and OFDR, FBG and OFDR have been widely used in the measurement of temperature, strain, stress and other physical parameters [13,14,15]. As the basic application of optical fiber sensing technology, strain measurement, as a key index, needs further calibration and analysis.

As an elastic sensitive element, the beam with equal strength has the advantages of structural stability and convenient processing, and it is an isosceles triangle in shape. When the vertex of the triangle is subjected to concentrated load, the free end changes in deflection, and the stress and strain on the section at any distance from the vertex are constant. It is commonly used in various stress and strain detection tests.

In this paper, the standard Beams of equal strength calibration system with mature the Electric Strain Gauge (ESG) and two fiber optic sensing technologies, FBG and OFDR, was used to carry out accuracy calibration experiments and related analysis. After several Beams of equal strength loading and unloading tests, the fiber-optic single-point strain data with the Beams of equal strength loading and unloading changes showed good continuity and linearity, and the cyclic stability was better. The error of strain test data was less than 2% after repeated loading.

To accurately describe the strain distribution and numerical accuracy of the Beams of equal strength, a finite element simulation model was used to compare and analyze with the measured data, which showed an error of less than 5% and good linearity of the respective strain test curves. As the number of loading and unloading cycles increased, the local strain jumps in the ESG measurement data became more prominent, and the error of the strain test data was less than 4% after repeated loading. After repeated loading and unloading tests, it was verified that the fiber grating and distributed fiber exhibited good stability in terms of the repeatability accuracy of the strain test, while the strain gauges exhibited local strain shifts after multiple repeated loading and unloading cycles. This experiment further verified that OFDR distributed fiber optic sensing technology, FBG, and ESG were essentially comparable in terms of strain test accuracy, and also validated the high repeatability stability of fiber optic sensing technology. The calibration method provided technical support for the basic application of fiber optic sensing technology.

## 2. Strain Measurement Principles

### 2.1. Principle of ESG Measurement Technology

**Figure 1:**
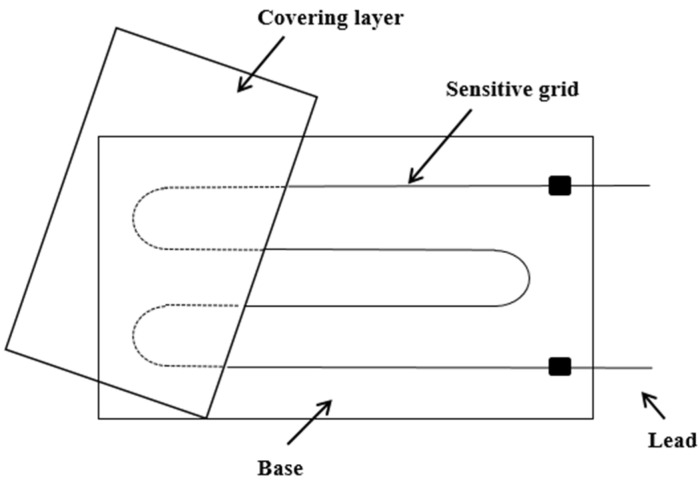
Structure diagram of resistance strain gauge.

Strain gauge is composed of five parts: sensitive grid, substrate, cover layer, adhesive and lead wire. As shown in Figure 1, the sensitive grid is the core part of the resistance strain gauge, and its role is to convert the strain change into its own resistance change. The lead wire connects the strain gauge to the test device, which can transfer the electrical signal collected by the sensitive grid to the detection device. By attaching the resistance strain gauges to the surface of the structure, the strain in the structure can be converted into a change in the resistance of the strain gauges.

Metal strain gauge resistance sensors mainly work by utilizing the strain effect of resistance, i.e., the amount of change in the resistance value of the metal sheet in the strain gauge is used to measure the small deformation caused by the applied external force or by the external force.

(1) $$\frac{dR}{R}=K_{0}\varepsilon$$

*R* is the initial resistance of the metal wire, as seen in Equation (1), the resistance of the resistance wire is subjected to force, the rate of change of its resistance is proportional to the strain produced by the resistance wire, which is the working principle of the strain gage [16]. *K*_0_ is called the sensitivity coefficient of the resistance wire, and its value is a constant in the proportionality limit of the stretching of the resistance wire. *K*_0_ is generally determined experimentally, and the *K_0_* for Conoco is generally 1.9–2.1, and that of Chromium is generally 2.1–2.3.

### 2.2. FBG Measurement Technology Principle

**Figure 2:**
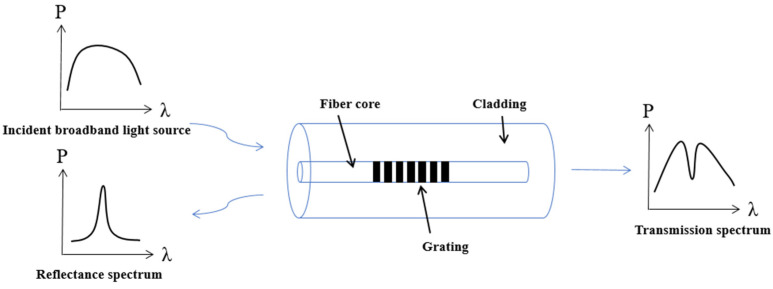
Schematic diagram of FBG structure.

Fiber grating is essentially a process of periodically changing the refractive index of a section of optical fiber within the core of optical fiber, as shown in Figure 2 below. Under normal circumstances through the light will all pass through the Bragg grating without being affected, only a specific wavelength of light in the Bragg grating will be reflected back to the original direction, equivalent to a narrowband reflector or filter. The FBG obtains the variation of the physical quantity to be measured by monitoring the drift of the grating wavelength. The expression for the FBG wavelength is [17]:

(2) $$\lambda ={2n}_{\mathrm{eff}}\Lambda$$

where *λ* is the center wavelength of the FBG, *n_eff_* is the effective refractive index of the fiber core, and ∧ is the grating period. When the FBG is located in the environment temperature or stress changes, external forces will lead to *n_eff_* or ∧ change, which will cause the movement of the FBG center wavelength. The FBG strain sensing characteristic expression is:

(3) $$\Delta \lambda_{B}=\left\{1-\left[P_{12}-\left(P_{11}+P_{12}\right)v\right]\frac{{n^{2}}_{\mathrm{eff}}}{2}\right\}\lambda_{B}\varepsilon_{Z}=K_{\varepsilon }\lambda_{B}\varepsilon_{Z}$$

where $\lambda_{B}$ is the strain sensing sensitivity coefficient, for ordinary quartz fiber, the elastic optical coefficient *P*_11_ = 0.121, *P*_12_ = 0.27, *V* is the Poisson’s ratio of fiber grating, *V* = 0.17, *n_eff_* = 1.4438.

### 2.3. OFDR Demodulated Distributed Fiber

**Figure 3:**
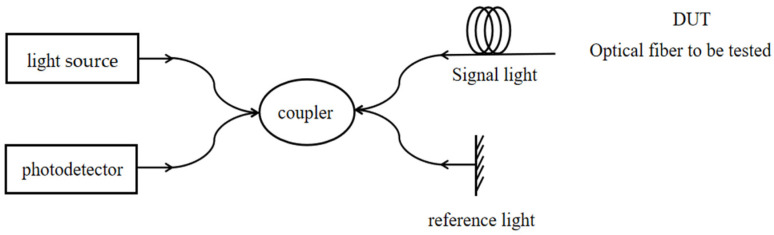
Basic principle of OFDR demodulation technology.

The optical fiber manufacturing process has a specific Rayleigh scattering spectral distribution due to uneven refractive index distribution. A certain position of the fiber is affected by temperature or strain, causing a shift in the Rayleigh scattering frequency, where there is a linear relationship between the amount of frequency shift and temperature and strain [13]. OFDR is an optical frequency domain reflection technology, which is combined with optical heterodyne detection technology to locate the scattered signal by measuring the frequency of Rayleigh scattering signal in optical fiber. As shown in Figure 3, the linear scanning light emitted by the light source is divided into two paths of light through the coupler, in which one path of light wave is injected into the sensing fiber, and when it propagates in the fiber, it will continuously generate Rayleigh scattering signals, which become signal light and are coupled into the detector through the coupler, and the other path of light wave is also coupled into the detector through the coupler as reference light after reflection, and the relationship curve between frequency and Rayleigh scattering intensity is located through the optical heterodyne coherent detection technology, and then the frequency domain is converted into the time domain through Fourier transform [18].

**Figure 4:**
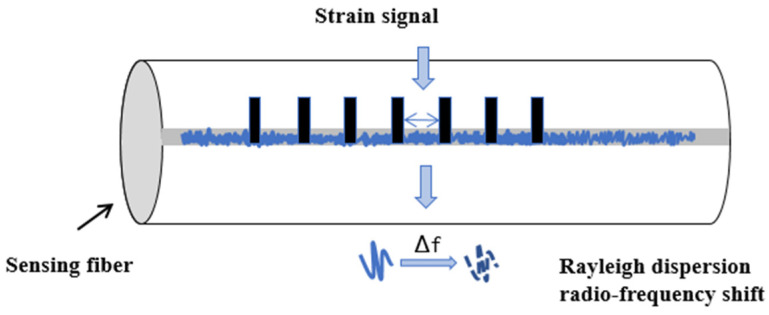
OFDR sensing test principle.

When the optical fiber is affected by external temperature and stress, its frequency spectrum will drift. By calculating the frequency shift coefficient of temperature and stress, the temperature and strain changes at this position can be obtained, and the fully distributed sensing of temperature and strain sensing can be realized. The principle of OFDR sensing test is shown in Figure 4 below.




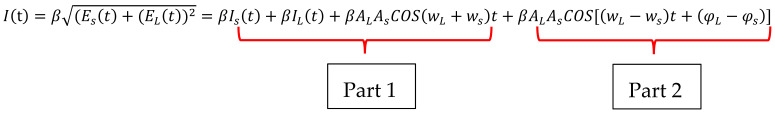

(4)



Part 1: DC and high frequency terms are filtered out.

Part 2: Beat frequency term measured by detector The intensity correspond to that return loss and reflectivity of the optical signal in the optical fiber. The distance corresponding to the beat frequency is used for positioning along the optical fiber.

A linear sweep laser combined with coherent detection technology can detect the Rayleigh backscattering signal in the optical fiber and obtain the Rayleigh scattering information of the whole fiber. It is suitable for distributed optical fiber sensing with short distance, high resolution, and high precision. Its spatial resolution is at the millimeter level, and its measurement range can reach 100 m. The distribution result of the whole fiber can be theorized and measured, and the local difference in strain parameters in the target area can be obtained by a single measurement.

Along the length of the fiber, the fiber to be tested is divided into one adjacent sensing unit at equal intervals, demodulate the frequency shift of the Rayleigh scattering spectral signals before and after the loading of each sensing unit, and then combine the frequency shift with the strain temperature conversion coefficient to derive the strain value. All the sensing units of the whole fiber are calculated one by one, and the strain distribution with distance can be obtained.

## 3. Experimental Setup

**Figure 5:**
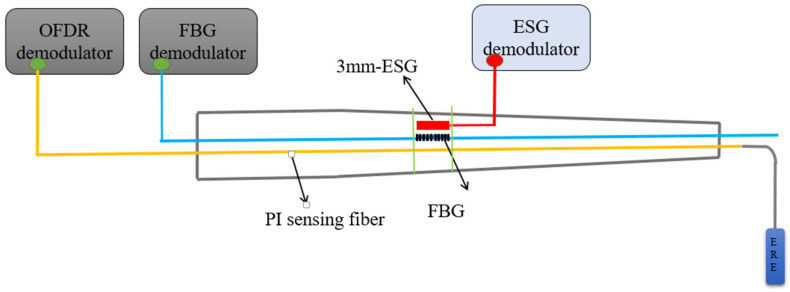
Schematic diagram of sample deployment.

In the experiment, three types of sensors were sequentially deployed onto an isostress beam for testing, as illustrated in Figure 5, which shows the schematic diagram of the sample deployment. Equal strength beam means that the maximum normal stress on each cross section of the beam is equal and reaches the allowable stress of the material. The material of equal strength beam used in this experiment is aluminum alloy, and the working size is *L × B × H* = 295 × 9.25 × 3 mm. The strain calculation formula for beams with equal strength is:

(5) $$\varepsilon =\frac{\sigma }{E}\frac{P}{H^{2}}\frac{l}{B}$$

where *L*, *H* and *E* are the length, thickness and elastic modulus of beams with equal strength, respectively (*E* = 72 GPa). where *B* is the width of the load-bearing part and *σ* is the stress on the beam section. Tensile stress, p is the acting force on the beam with equal strength.

**Figure 6:**
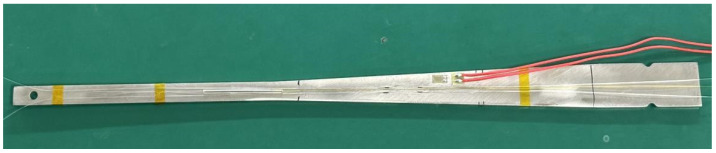
Actual sensor image of sample deployment.

Among them, the Fiber Bragg Grating (FBG) system utilized a fiber optic grating with a central wavelength of 1565 nm as the sensor, the Optical Frequency Domain Reflectometry (OFDR) system employed a bend-insensitive polyimide optical fiber, and the Electric Strain Gauge (ESG) was equipped with a sensor featuring a sensitive grid length of 3 mm, as depicted in the actual sensor image shown in Figure 6. In this test, a fiber grating with a central wavelength of 1565 nm, a strain gauge with a sensitive grating length of 3 mm and a single-mode polyimide fiber with a length of 20 cm are used as strain test sensors. The experimental process involved loading weights incrementally, with each increment consisting of a 100 g weight, totaling 15 levels of loading.

**Figure 7:**
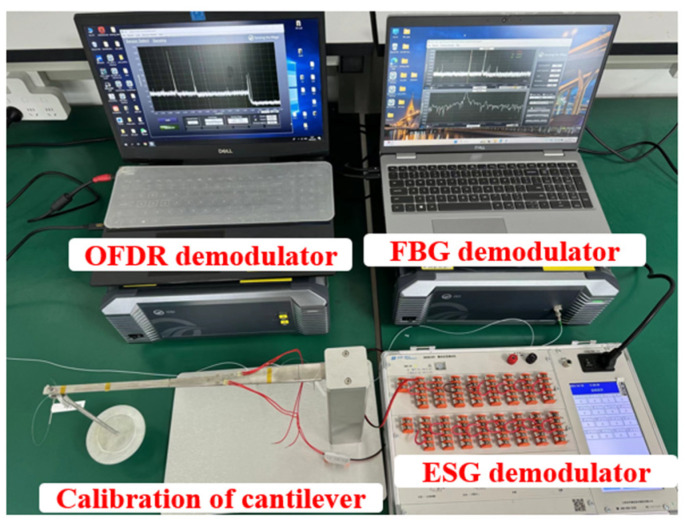
Strain calibration system based on fiber optic sensing technology.

In this experiment, the strain gauge demodulator is DH3818Y static stress and strain test and analysis system produced by Jiangsu Donghua Test Technology Co., Ltd., (Jingjiang, China) with a strain range of ±10,000 με, a resolution of ±0.1 με and a static sampling rate of 5 Hz. The fiber grating demodulator is OCI series equipment produced by Wuhan Megasense Technologies Co., Ltd. (Wuhan, China). The strain and temperature measurement is realized by calculating the center wavelength offset of grating. The strain range is ±4000 με, the spatial resolution is in the nanometer level, and the demodulation rate is 2 kHz. OFDR system uses OSI series equipment produced by Wuhan Megasense Technologies Co., Ltd. (Wuhan, China). The OFDR system uses a narrow-band linear swept-frequency light source, with a working wavelength of 1530–1570 nm, a strain range of ±12,000 με, a spatial resolution of 1mm, a maximum sampling rate of 100 Hz and a strain accuracy of ±1 με. The above three sensing demodulation devices are shown in Figure 7.

During the experiment, the isostress beam’s state without any weights placed was recorded as the zero state. The weights were incrementally added by 100 g, and the strain test data for each stable state after loading were recorded. For data recording, a grating demodulator, a strain demodulator, and an OFDR device were used sequentially to record and save the strain test data for the grating, strain gauge, and distributed optical fiber strain tests.

## 4. Results and Discussion

**Figure 8:**
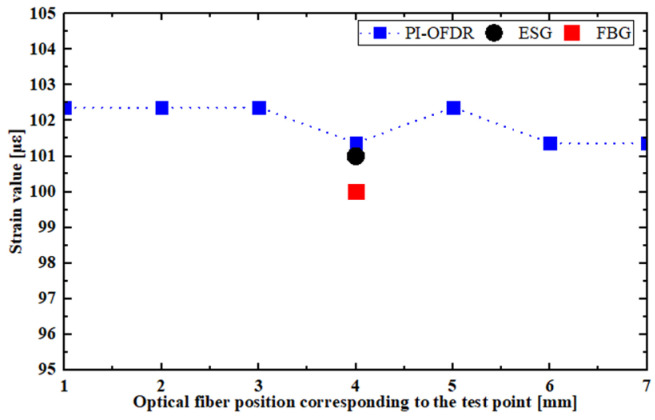
Distribution of strain test data for FBG, ESG, and PI-OFDR.

When loading with a 200 g weight, as depicted in Figure 8, strain test data from the three types of sensors were obtained. It can be observed that both the strain gauge and the grating, after being demodulated by the demodulator, output a single strain value, whereas the strain test data from the distributed optical fiber is continuously distributed. The strain value measured by the strain gauge represents the average strain data within its sensitive grid section, and the strain value measured by the grating is determined by the strain size caused by the central wavelength shift within its grating area. Therefore, during data processing, it is necessary to average all the strain data from the sensing points within the optical fiber section corresponding to the sensitive grid of the strain gauge. This average is then used as the measurement result for the optical fiber, which is compared with the measurement data from the fiber grating and the strain gauge.

**Figure 9:**
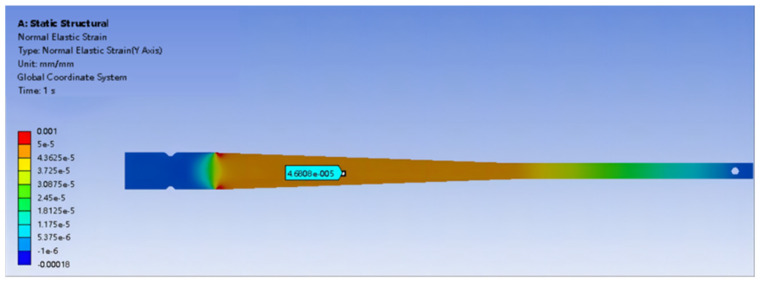
Strain generated by adding a 100 g weight to the equi-strength Beam.

**Figure 10:**
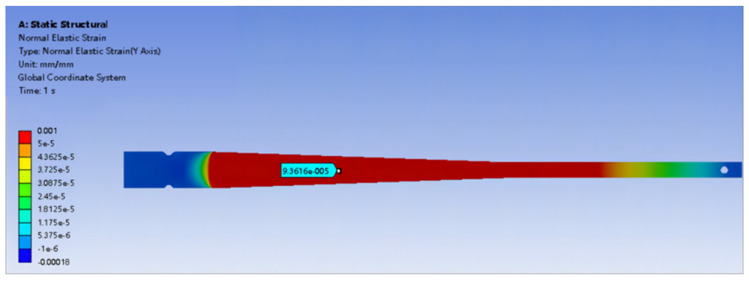
Strain generated by adding a 200 g weight to the equi-strength Beam.

Finite element analysis was conducted on the equi-strength beam used in the experiment. At the same location on the equi-strength beam, a strain increase of 47 micro-strain (με) was observed for every 100 g weight added, as shown in Figure 9 and Figure 10.

**Figure 11:**
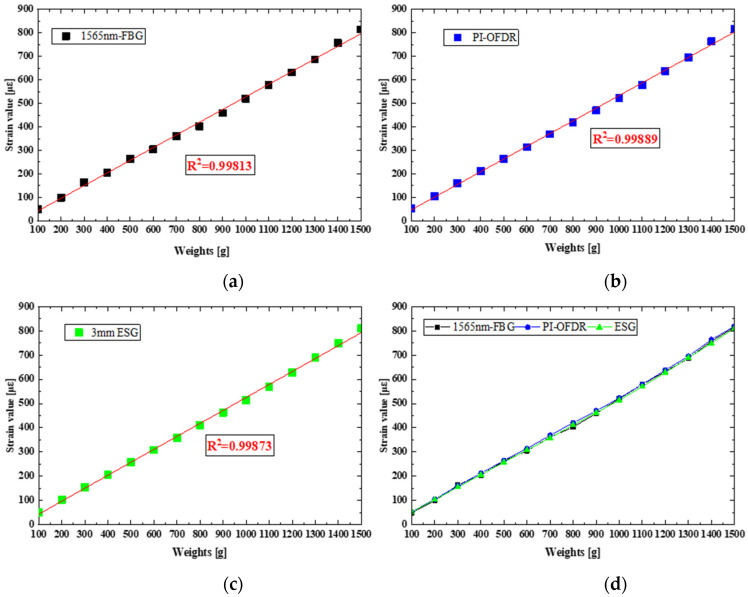
Comparison of strain test data. (**a**) Load strain test data for each stage of FBG; (**b**) Load strain test data for each stage of PI-OFDR; (**c**) Load strain test data for each stage of ESG; (**d**) Comparison of strain distribution among the three sensors.

Table 1 shows the test loading data, with 100 g weights per stage and 15 stages in total. Three groups of test data are recorded, and the average value is plotted as shown in Figure 11, in which the horizontal axis represents the increased weight and the vertical axis represents the measured strain value. The strain test data from three sensors are fitted linearly in turn, and the fitting coefficients (r) are 0.99813, 0.99889 and 0.99873 respectively. The good linear relationship shows that the test data in this experiment is accurate and effective. When comparing the strain test data from 1565 nm fiber grating, distributed fiber and 3 mm strain gauge, the error is less than 2%. Compared with the data of finite element theory analysis, the error between the measured data and the data of finite element theory analysis is less than 5%.

**Figure 12:**
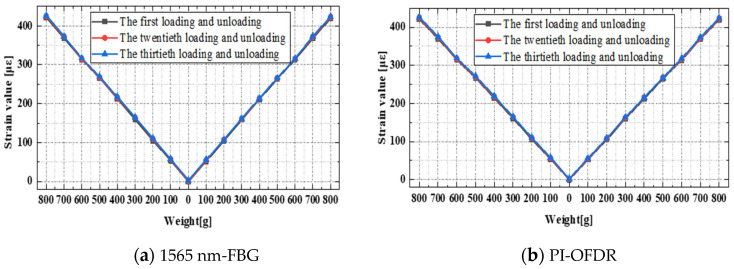
Strain data of FBG and PI-OFDR after multiple loading and unloading cycles.

**Figure 13:**
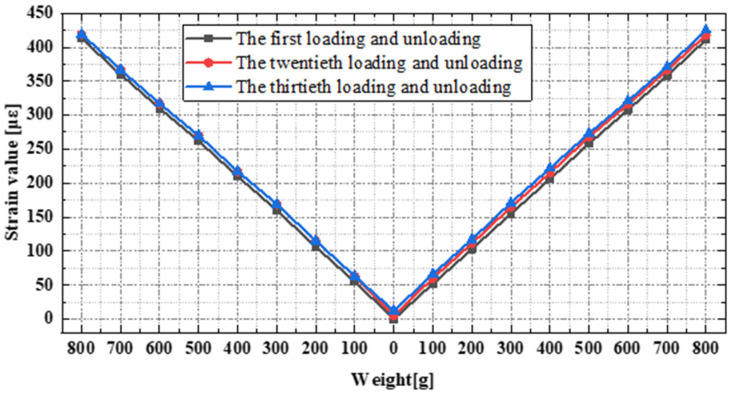
Strain data of ESG after multiple loading and unloading cycles.

Multiple repeated loading and unloading cycles were conducted on the three sensors: the fiber grating, the distributed optical fiber, and the strain gauge. A 100 g weight was used for each loading/unloading stage, totaling eight stages. Based on the strain test results from the three sensors as depicted in Figure 12 and Figure 13, the following conclusions can be drawn. The single-point strain data from the fiber grating and the distributed optical fiber exhibited good continuity and linearity with changes in loading and unloading, demonstrating better cyclic stability. After repeated loading, the strain test data error was less than 2%. In contrast, the strain gauge measurements showed increasingly noticeable local strain jumps with an increasing number of loading and unloading cycles. After multiple repeated loading cycles, the strain test data error was less than 4%. This, apart from being attributed to the intrinsic performance of the strain gauge, may also be related to the adhesive properties of the strain gauge. The adhesive layer between the sensitive grating of the strain gauge and the substrate, and the gluing between the substrate and the test piece will cause the strain gauge data to drift when they are displaced and degummed.

It can be clearly seen from Figure 13 above that the strain data measured by strain gauge began to drift obviously after repeated loading of the test piece for many times. Under normal circumstances, there are two kinds of situations that lead to the deviation of test data: 1: Repeated loading causes irreversible damage to the test piece itself, which leads to data drift; 2: The stability of repeated loading test of the sensor itself is weak, and some irreversible strain remains in the sensor itself after repeated loading. Through the loading test of the equal strength beam specimen alone, after repeated loading tests, the aluminum alloy specimen of the equal strength beam is in good structural condition without damage, so the deviation of the strain gauge data caused by the specimen itself is ruled out, which further ensures the reliability and accuracy of the test.

In order to avoid the contingency of the selected loading data, the test data are statistically processed again and plotted as shown in Table 2. The FBG, PI and ESG sensors are repeatedly loaded with 800 g weight, and an average value is taken every 40 groups of data, and the error rate of strain test data after repeated loading is analyzed and calculated.

## 5. Conclusions

This paper presents precision calibration experiments and related analyses using a standardBeams of equal strength system equipped with a mature ESG, along with two types of fiber optic sensing technologies: FBG and OFDR. After multipleBeams of equal strength loading and unloading tests, it was found that the fiber optic single-point strain data exhibited good continuity and linearity with changes in loading and unloading, and better cyclic stability, with the error in strain test data after multiple repeated loading being less than 2%. Additionally, the finite element theoretical analysis data were within a 5% error margin of the actual measured data, indicating a good linearity of the strain test curves with R^2^ values greater than 0.998. Furthermore, repeated loading and unloading experiments demonstrated the good stability of FBG and OFDR in terms of strain testing repeatability and precision, while the strain gauge showed local strain shifts after multiple repeated loading and unloading.

The calibration experiments and data analysis in this paper validate the stability of OFDR distributed fiber optic sensing technology and FBG in strain testing accuracy, providing foundational technical support for the development of applications of fiber optic sensing technologies.

## Figures and Tables

**Table 1:** The trial loading data.

	Sensors	FBG/με				OFDR/με				ESG/με			
Load													
		First	Second	Third	Average	First	Second	Third	Average	First	Second	Third	Average
100 g		48	51	48	49	52	54	53	53	51	54	51	52
200 g		98	101	98	99	106	104	104	105	104	102	103	103
300 g		164	163	166	164	159	160	161	160	155	156	154	155
400 g		206	204	202	204	212	211	213	212	204	205	209	206
500 g		262	264	263	263	265	266	264	265	255	259	260	258
600 g		306	303	306	305	315	313	314	314	310	306	308	308
700 g		359	365	359	361	368	369	370	369	357	359	358	358
800 g		403	402	404	403	422	420	418	420	413	411	409	411
900 g		457	461	459	459	471	469	470	470	459	463	463	462
1000 g		518	521	521	520	523	521	522	522	512	516	514	514
1100 g		577	580	580	579	579	580	578	579	570	569	571	570
1200 g		633	631	632	632	639	637	638	638	625	630	629	628
1300 g		685	687	689	687	695	694	696	695	689	690	688	689
1400 g		757	756	758	757	763	764	765	764	750	749	748	749
1500 g		818	814	813	815	819	820	815	818	810	809	811	810

**Table 2:** Single 800 g Weight Repeated Loading Test.

	Loading Times	Load 40 Times	Load 80 Times	Load 120 Times	Error Rate
Sensors					
FBG		420 με	424 με	426 με	<2%
PI		419 με	425 με	427 με	<2%
ESG		425 με	432 με	441 με	<4%

## Data Availability

The data presented in this study are available on request from the corresponding author. The data are not publicly available due to privacy concerns.
